# Serotonylation of Vascular Proteins Important to Contraction

**DOI:** 10.1371/journal.pone.0005682

**Published:** 2009-05-25

**Authors:** Stephanie W. Watts, Jessica R. C. Priestley, Janice M. Thompson

**Affiliations:** Department of Pharmacology and Toxicology, Michigan State University, East Lansing, Michigan, United States of America; Abramson Research Center, United States of America

## Abstract

**Background:**

Serotonin (5-hydroxytryptamine, 5-HT) was named for its source (sero-) and ability to modify smooth muscle tone (tonin). The biological effects of 5-HT are believed to be carried out by stimulation of serotonin receptors at the plasma membrane. Serotonin has recently been shown to be synthesized in vascular smooth muscle and taken up from external sources, placing 5-HT inside the cell. The enzyme transglutaminase uses primary amines such as 5-HT to covalently modify proteins on glutamine residues. We tested the hypothesis that 5-HT is a substrate for transglutaminase in arterial vascular smooth muscle, with protein serotonylation having physiological function.

**Methodology/Principal Findings:**

The model was the rat aorta and cultured aortic smooth muscle cells. Western analysis demonstrated that transglutaminase II was present in vascular tissue, and transglutaminase activity was observed as a cystamine-inhibitable incorporation of the free amine pentylamine-biotin into arterial proteins. Serotonin-biotin was incorporated into α -actin, β-actin, γ-actin, myosin heavy chain and filamin A as shown through tandem mass spectrometry. Using antibodies directed against biotin or 5-HT, immunoprecipitation and immunocytochemistry confirmed serotonylation of smooth muscle α–actin. Importantly, the α-actin-dependent process of arterial isometric contraction to 5-HT was reduced by cystamine.

**Conclusions:**

5-HT covalently modifies proteins integral to contractility and the cytoskeleton. These findings suggest new mechanisms of action for 5-HT in vascular smooth muscle and consideration for intracellular effects of primary amines.

## Introduction

The primary amine 5-hydroxytryptamine (5-HT, serotonin) is a hormone which exerts multiple effects in the vasculature, including vasoconstriction, vasodilation, endothelial and smooth muscle cell mitogenesis, and potentiation of contractile and mitogenic effects of vasoactive hormones [1], [2]. Multiple 5-HT receptor families (5-HT_1_–5-HT_7_) and subtypes exist, and it is through stimulation of these receptors to which the biological actions of 5-HT have been attributed [3]. Recent evidence suggests that the role of 5-HT in the vasculature is more complex than previously appreciated.

We recently discovered that a serotonergic system exists in systemic arteries [4]. Systemic arteries, including the superior mesenteric artery and thoracic aorta, can synthesize 5-HT, metabolize 5-HT to 5-hydroxyindole acetic acid (5-HIAA), take up and release 5-HT. Thus, there are at least two mechanisms by which 5-HT can be placed inside a cell, the first through synthesis and the second through uptake of circulating 5-HT by the serotonin transporter [5]. The existence of intracellular 5-HT raises the question as to the function of 5-HT inside the cell. Serotonin was recently shown to covalently modify small GTPases in the platelet [6]. In this paper, the enzyme transglutaminase (TG) placed 5-HT on glutamine residues of small GTPases to form a glutamyl-amide bond (serotonylation), resulting in activation of the G protein. The platelet, however, is a cell that is enriched in 5-HT (mM concentration), leading to the question as to whether serotonylation was relevant to a cell in which 5-HT was not highly concentrated. More recently, serotonylation of Rho in the pulmonary artery was demonstrated, but this again is a tissue exposed to and which clears significant concentrations of 5-HT [7], [8].

We hypothesized that 5-HT would covalently modify systemic arterial proteins by acting as a substrate for TG, and that this process was physiologically relevant. Our model was the aorta of the rat as this blood vessel contracts to 5-HT, possesses a complete serotonergic system and the receptor mechanisms of contraction are known [5-HT_2A_ receptor-mediated contraction; 9]. Important to these experiments was synthesis of a biotin-conjugated 5-HT that allowed us to identify and track proteins that were serotonylated. We discovered serotonylation of proteins important to contraction and cell shape, and that this may have physiological significance.

## Materials and Methods

### Animal use/Ethics Statement

Male Sprague-Dawley rats (250–300 g; Charles River Laboratories, Inc., Portage, MI, USA) were used. Rats were anesthetized with pentobarbital (60 mg kg^−1^, i.p.) prior to removal of tissues. Procedures that involved animals were performed in accordance with the guidelines of *Michigan State University*, and approved by the Institutional Animal Use and Care Committee.

### Immunohistochemistry

Paraffin-embedded tissue sections were dewaxed, unmasked and taken through a protocol as previously described [4]. Primary antibodies used were: transglutaminase II (TGII; mouse monoclonal TG100, LabVision, Fremont CA, USA), N-epsilon gamma glutamyl lysine (mouse monoclonal, ab424, Abcam, Cambridge, MA, USA). In some experiments, primary antibodies were left out of the experiment, and tissues developed only in the presence of secondary antibody.

### Western analysis

Protein isolation and western blotting procedures were performed as previously described [4] using standard SDS-PAGE conditions and blotting proteins to nitrocellulose. Primary antibodies used were TGII (LabVision, Fremont CA, USA), smooth muscle cell α-actin (mouse monoclonal, Ab-2, EMD Biosciences, La Jolla, CA, USA) and 5-HT (AbD Serotec, Raleigh NC, USA). Films were scanned and placed within the figure without gamma modifications using Adobe Photoshop.

### Transglutaminase activity

Protein homogenate (50 µg) was placed in transglutaminase reaction buffer (50 mM Tris-HCl, pH 7.5, 150 mM NaCl, 1 mM phenylmethylsulfonyl fluoride, 2 mg/ml aprotinin and leupeptin, 1 mM sodium orthovanadate, 5 mM calcium chloride) containing pentylamine biotin (BAP; 8 mM) or biotinylated amine (IBL, Hamburg, Germany). For biotinylation of 5-HT, 5-HT hydrochloride (Sigma Germany) and EZ-Link Sulfo-NHS-LC-LC-Biotin (Pierce) were used. Equimolar amounts of serotonin and NHS-LC-LC-Biotin were used with pyridine as solvent. After a period of several hours on a roller mixer, the turbid solution was stored over night. On next morning, solvent was evaporated in a vacuum centrifuge and the residue re-suspended in dimethylformamide (DMF). The coupling of the indole amine to biotin was checked through 5-HT enzyme immunoassay (ELISA) by running the ELISA according to manufacturer instructions (IBL International GmbH, Hamburg, Germany Serotonin ELISA cat. No. RE59121). Instead of Biotin required in the *Instructions for Use* included in the kit, freshly synthesized biotinylated serotonin in different dilutions was used. The purity was checked greater than 90%. Stock concentration was 1.59 mM.

Amines were incubated in the presence of vehicle or the TGII inhibitor cystamine (0.001–10 mM) at 37°C for one hour. An equal volume of 2× SDS sample buffer was added to stop the reaction and the samples were boiled for 10 minutes. Samples were separated on 10% polyacrylamide gels (Bio Rad CA, USA), and transferred to nitrocellulose. Samples were blocked overnight at 4°C in 4% chick egg ovalbulmin [TBS-0.1% Tween+0.025% NaN_3,_], washed in TBS-Tween for 20 minutes, and incubated with streptavidin-linked, horseradish peroxidase-conjugated secondary antibody (1∶2000, 1 hr, 4°C GE Healthcare, Piscataway NJ, USA). ECL® reagents (GE Healthcare, Piscataway NJ USA) were used to visualize bands. Films were scanned and placed within the figure without gamma modifications using Adobe Photoshop.

### 5-HT measurement

At room temperature, dissected and cleaned aorta were placed in 100 µL physiological salt solution [PSS: 103 mM NaCl; 4.7 mM KCl; 1.18 mM KH_2_PO_4_; 1.17 mM MgSO_4_-7H_2_O; 1.6 mM CaCl_2_-2H_2_O; 14.9 mM NaHCO_3_; 5.5 mM dextrose, and 0.03 mM CaNa_2_ EDTA]. Tissues were briefly dipped in fresh PSS and placed in tissue buffer (0.05 mM sodium phosphate and 0.03 mM citric acid buffer (pH 2.5) containing 15% methanol]. Tissue samples were frozen in −80°C until assay. Samples were thawed, sonicated for 3 seconds and centrifuged for 30 seconds (10,000 g). Supernatant was collected and transferred to new tubes. Tissue pellets were dissolved in 1.0 M NaOH and assayed for protein (Lowry assay). Concentrations of 5-HIAA and 5-HT in tissue supernatants were determined by isocratic high pressure liquid chromatography (HPLC/ESA; described below).

### Cell culture and 5-HT uptake

Aortic cells were derived from explants of thoracic aorta. Cells were fed with DMEM supplemented with 10% fetal bovine serum and 1% penicillin/streptomycin. Cells were plated to P-60 dishes or coverslips for Western and immunocytochemical experiments, respectively. Cells used were between passages 2 and 9 and all explants stained positive for smooth muscle cell α-actin (EMD Biosciences, La Jolla, CA, USA). Cells were starved of serum 24 hours prior to experimentation because serum contains 5-HT (determined by HPLC). Cells were incubated for 1 hour in PSS and (+/−10 µM pargyline, a monoamine oxidase A inhibitor) with vehicle (0.01% DMSO) or fluoxetine (1 µM) prior to addition of 5-HT (10^−8^–10^−5^ M) or 5-HT-biotin (0–12.7 µM). Cells were placed on ice, washed with PSS, scraped in tissue (HPLC analyses) or lysis buffer (Western analyses), and centrifuged (14, 000 rpm, 10 minutes). The supernatant was removed for HPLC or Westerns (described above). Protein concentration of supernatant and pellet were measured using the Lowry assay.

### HPLC measurement

Samples were thawed, sonicated for 3 seconds and centrifuged for 30 seconds (10,000 g). Supernatant was collected and transferred to new tubes. Tissue pellets were dissolved in 1.0 M NaOH and assayed for protein. Concentrations of 5-HIAA, 5-HTP and 5-HT in tissue supernatants were determined by isocratic high pressure liquid chromatography (HPLC/ESA Systems) using electrochemical detection. An ESA MD-150 C18 column was used at 0.4 V and 0.6–9 ml/min flow rate (mobile phase: 90 mM NaH_2_PO_4_, 50 mM citric acid, 1.7 mM 1-octanesulfonic acid, 50 µM EDTA, 10% acetonitrile) as compared to standards run daily. Data are reported as ng amine/ mg protein.

### Immunocytochemistry

Cells adherent to cover slips were equilibrated in PSS 30 minutes (+10 µM pargyline) prior to a one hour incubation with 5-HT-biotin (12.7 µM) or 5-HT (10 µM) and vehicle or cystamine (10 mM). Cells were rinsed and fixed in 1 mL acetone (1 minute). Primary antibody used was anti-α-actin, smooth muscle specific (mouse monoclonal, Ab-2, EMD Biosciences, La Jolla CA, USA 1∶100 in PBS) or anti-5-HT (rabbit polyclonal, 8250-0004, AbD Serotec, Raleigh NC, USA). Secondary antibodies used were: DyLight™ 488 streptavidin (1∶2000, Rockland Inc, Gilbertsville, PA, USA) and Cy3-conjugated Affini Pure donkey anti-mouse (1∶1000; IgG, Jackson, West Grove PA, USA) or Cy3-conjugated Affini Pure donkey anti-rabbit (1∶1000; IgG, Jackson, West Grove PA, USA) in PBS. Following rinsing, cover slips were blotted dry and mounted on slides using Prolong Gold medium with DAPI (Invitrogen, Carlsbad, CA, USA). Slides were viewed and photographed on a Nikon TE2000 microscope using MetaMorph ® software (Molecular Devices, Sunnyvale CA, USA; all at 20°C). The light source was an X-Cite 120 fluorescence illumination system (EXFO, Mississauga Ontario, Canada), the camera a cool nap ES monochrome digital camera (Roper Scientific Photometrics, Pleasanton CA, USA). Slides were viewed under a 60× Nikon Plan Apo oil-immersion objective (Nikon Corporation, Toyko, Japan) using non-drying immersion oil, type a, formula code 1248 (Cargille, Cedar Grove, NJ, USA). At this magnification, 1 pixel is equivalent to 0.22 microns. Three Nikon filters were used: UV-2E/C96310M (lot number: C48793), Cy3 HYQ 96323 (lot number: C71280), and B-2E/C96311 (lot number: C57657). Excitation ranges were: UV-2E/C 340–380 nm, Cy5 HYQ 530–560 nm and B-2E/C465–495. Emission ranges were UV-2E/C435–C485, Cy3 HYA 573–648, and B-2E/C515–555 nM. Photograph bit depth was 12 nm. No neutral density filters or dichromic beamsplitters were utilized. The LUT was linear and covered the full range of data. Gamma values were equal to 1. Resolution of the photographs was 696×520 pixels (28.3 pixels/cm). There was no deconvolution, reconstruction, rendering or projection. Images were unaltered when combined into the overlay image.

### Cell Viability

Confluent petri dishes of rat aortic smooth muscle cells were rinsed of growth medium and equilibrated in physiological salt solution (PSS) for 30 minutes at 37°C and 5% CO_2_. Cells were incubated for 1 hour in 10 mM cystamine (Sigma-Aldrich, St. Louis, MO, USA) in PSS at 37°C and 5% CO_2_. The cystamine solution was gently removed but not rinsed from the dishes. Cells were incubated for 5 minutes with 0.05% trypsin-EDTA (Gibco, Invitrogen, Carlsbad, CA, USA). The cell/trypsin mixture was transferred to a conical tube and neutralized with 1.5 mL DMEM (Gibco, Invitrogen, Carlsbad, CA, USA) per 1 mL trypsin. This was centrifuged at 1400 RPM for 6 minutes. The supernatant was removed and the cell pellet was resuspended in 1 mL DMEM. Equal parts cell suspension and 0.4% trypan blue (Sigma-Aldrich, St. Louis, MO, USA) were mixed immediately before cell viability was ascertained using a Bright Line hemocytometer (Reichert, Buffalo, NY, USA) and Nikon TMS phase-contrast microscope (Nikon, Tokyo, Japan). Cells that took up the blue dye were deemed dead and percent viability was calculated by dividing the number of living cells (non-dye containing) by the total number of cells per square centimeter.

### Tandem Mass Spectrometry

Tissue homogenates were taken through a transglutaminase activity assay with and without 5-HT-biotin (12.7 µM). Samples incubated with streptavidin-coated magnetic beads (25 µL, Invitrogen, Carlsbad CA, USA) for 1 hour at room temperature, with tumbling. Samples were magnetized to pull down biotin-labelled proteins and supernatant discarded. The magnetic beads were boiled in 2× SDS loading buffer, and separated on polyacrylamide gels (10%). Bands were excised and taken through tandem mass spectrometry by the proteomics core at *Michigan State University*. Those bands reported are those that only appeared in 5-HT-biotin-labelled samples.

### Immunoprecipitation

Immunoprecipitation was carried out as previously described [9], using primary antibody against smooth muscle cell α-actin [(1 µg/200 µg protein) EMD Biosciences, La Jolla, CA, USA] in a phosphate-buffered saline based buffer. Samples were incubated overnight with protein A/G beads (25 µl/sample, Santa Cruz Biotechnologies, Santa Cruz, CA, USA), washed 3× with a protease-inhibitor rich phosphate buffered saline and then beads spun down. Captured beads were incubated in 2× SDS sample buffer, boiled for 10 minutes, centrifuged and the supernatant loaded onto standard SDS-PAGE gels (10%), at which point standard western protocol was used.

### Isometric contraction

Helical strips of endothelial cell-intact strips were mounted in tissue baths for isometric tension recordings using Grass transducers and PowerLab data Acquisitions (Colorado Springs, CO, USA). Strips were placed under optimum resting tension (1500 milligrams) and equilibrated for one hour, with washing, before exposure to compounds. Tissue baths contained warmed (37°C), aerated (95% O_2_/CO_2_) PSS. Administration of an initial concentration of 10 µM phenylephrine (PE) was used to test arterial strip viability. All tissues had an intact endothelial cell layer, evidenced by a robust (>50%) relaxation to acetylcholine (1 µM) in tissues contracted with a half-maximum concentration of PE. Tissues were incubated for one hour with vehicle (water) or cystamine (0.1–1 mM) for one hour prior to cumulative addition of 5-HT (10^−9^–10^−5^ M) or the non-receptor mediated agonist potassium chloride (KCl, 6–100 mM). Data are reported as the percentage of the initial contraction to PE [9].

### Materials

All compounds were purchased from Sigma Chemical Company (St. Louis, MO, USA) unless otherwise noted.

### Statistical analyses

All values are reported as means±standard error of the mean for the number of animals or explants (N) indicated. Data were analyzed by ANOVA with repeated test where more than two groups were compared (Graph Pad Prism), or two-tailed t test when two groups were compared. P values smaller than 0.05 were considered significant.

## Results

### Transglutaminase is present and functional in aortic tissue

**Figure 1:**
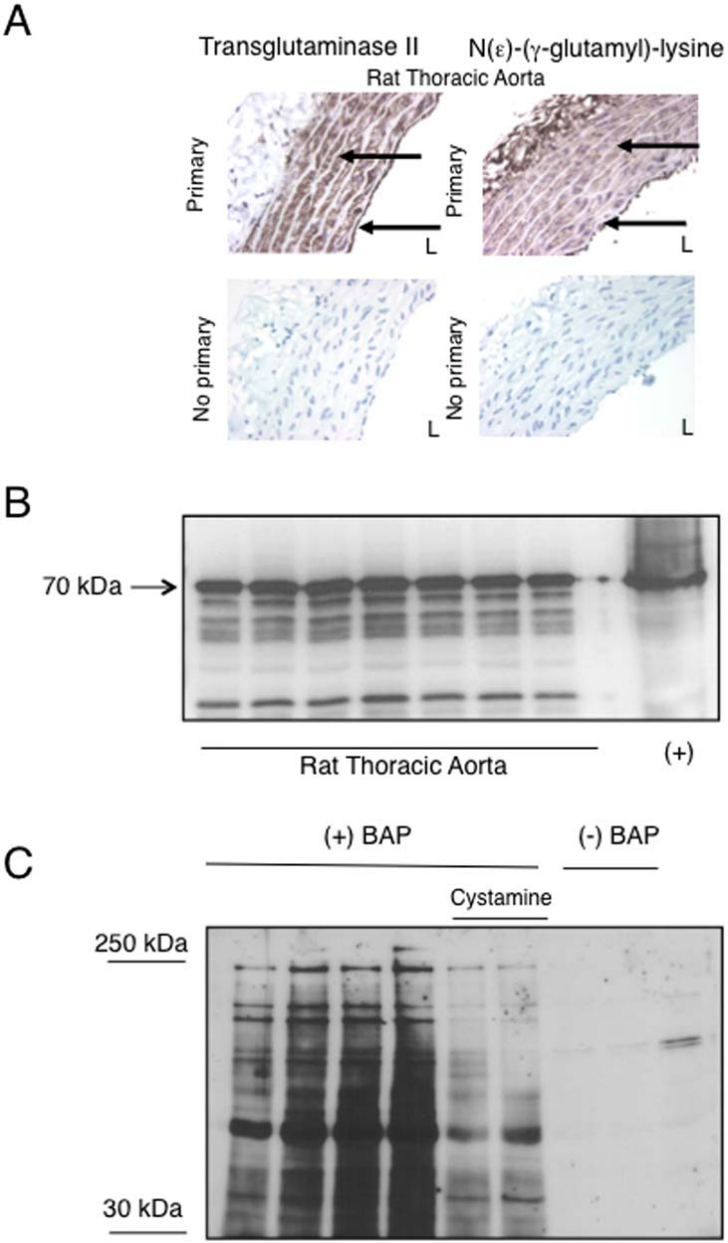
TG protein and activity are observed in rat aorta. A. Immunohistochemical localization of TG II and the classical protein product containing an N(ε)-(γ-glutamy)-lysine in band in normal rat thoracic aorta. Arrows point to positive staining that is present in samples incubated with primary antibody (primary) but is lost when the primary antibody is removed from the reaction (no primary). Representative of four (4) separate animals. L = lumen. B. Western analysis demonstrating the presence of TGII in homogenate of the rat aorta (arrow). Each lane represents a different animal. Positive control is the rat liver. C. TG activity assay in homogenate from rat thoracic aorta. Samples were incubated in normal TG buffer (in the absence and presence of the TG substrate BAP (−BAP, +BAP respectively), or TG inhibitor cystamine (10 mM). Representative of N>18.

**Figure 1A** shows immunohistochemical localization of TG II to the smooth muscle layers of the media in the aorta (lying between elastin/collagen cables), and, to a lesser extent, localization of the classical protein product of TG, N(ε)-(γ-glutamyl) lysine bonds. Using the same TG antibody, a robust band of the appropriate molecular weight (∼70 kDa) was identified in aortic homogenate (**figure 1B**). It is unclear whether the bands smaller in size but recognized by the TG antibody are degradative products. **Figure 1C** demonstrates that the TG present in aortic homogenates is functionally active as proteins readily incorporate the biotin-linked amine donor pentylamine biotin (+BAP), compared to samples that had no amine donor (−BAP). Protein modification was significantly reduced by incubating samples with the TG inhibitor cystamine, observed as a decrease in intensity of bands. Cystamine concentration (1–10 mM) was chosen, as in preliminary experiments a concentration response curve showed 0.001 mM–10 mM to have concentration-dependent inhibition of protein amination, with 1–10 mM cystamine causing maximal inhibition (data not shown).

### Endogenous 5-HT is present in arterial tissue and protein serotonylation is carried out by TGII

**Figure 2:**
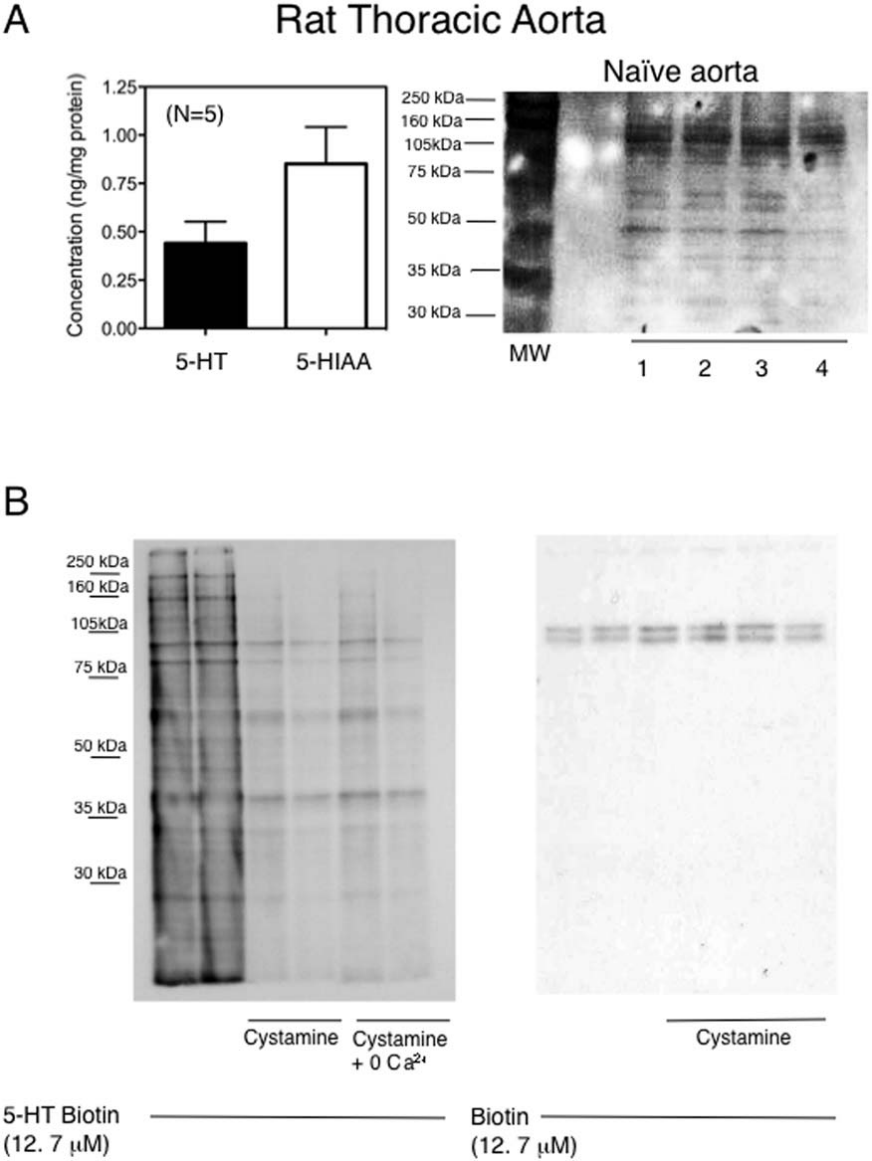
Endogenous 5-HT and TGII activity exists in rat aorta. A. Left: HPLC measurement of endogenous 5-HT and 5-HIAA in rat thoracic aorta for the number of animals indicated in parentheses. Bars and vertical lines represent means±SEM. Right: Recognition of proteins by an anti-5-HT antibody in normal rat aortic homogenate not exposed to the monoamine oxidase inhibitor pargyline or exogenous 5-HT. Each lane represents a separate animal, and this represents an N = 8. B. Left: TG activity assay using 5-HT-biotin as substrate in homogenates of rat thoracic aorta. Samples were incubated with or without the TG inhibitor cystamine (10 mM), as well as in the presence of cystamine in zero calcium TG buffer. Representative of N>30 different samples. Right: Lack of biotinylated proteins when biotin alone was used as a substrate in the absence or presence of cystamine. Each lane represents a different animal, representative of N = 8.

**Figure 2A** **(left)** demonstrates measurable amounts of 5-HT in freshly dissected rat aorta as detected by HPLC. The monoamine oxidase metabolite of 5-HT, 5-HIAA, was also present. The possibility of endogenous protein being serotonylated is supported by the protein bands recognized by an anti-5-HT antibody in homogenates of normal rat aorta not exposed to pargyline or exogenous 5-HT (**figure 2A** **, right**). **Figure 2B** **(left)** demonstrates that when 5-HT, in its biotinylated form, was incubated with arterial homogenates, a significant number of proteins were labelled (*i.e.* serotonylated) in a cystamine (10 mM)-dependent fashion. Removal of calcium from the buffer (cystamine +0 Ca^2+^) did not further reduce protein serotonylation. When biotin alone was used at an equivalent concentration (12.7 µM), no incorporation of biotin into proteins could be observed **(** **figure 2B** **, right**). A narrow doublet at ∼90 kDa was observed, but this was not cystamine-inhibitable, indicating that this likely represents endogenous biotin.

### Protein serotonylation is time- and concentration-dependent, 5-HT_2A_ receptor-independent

**Figure 3:**
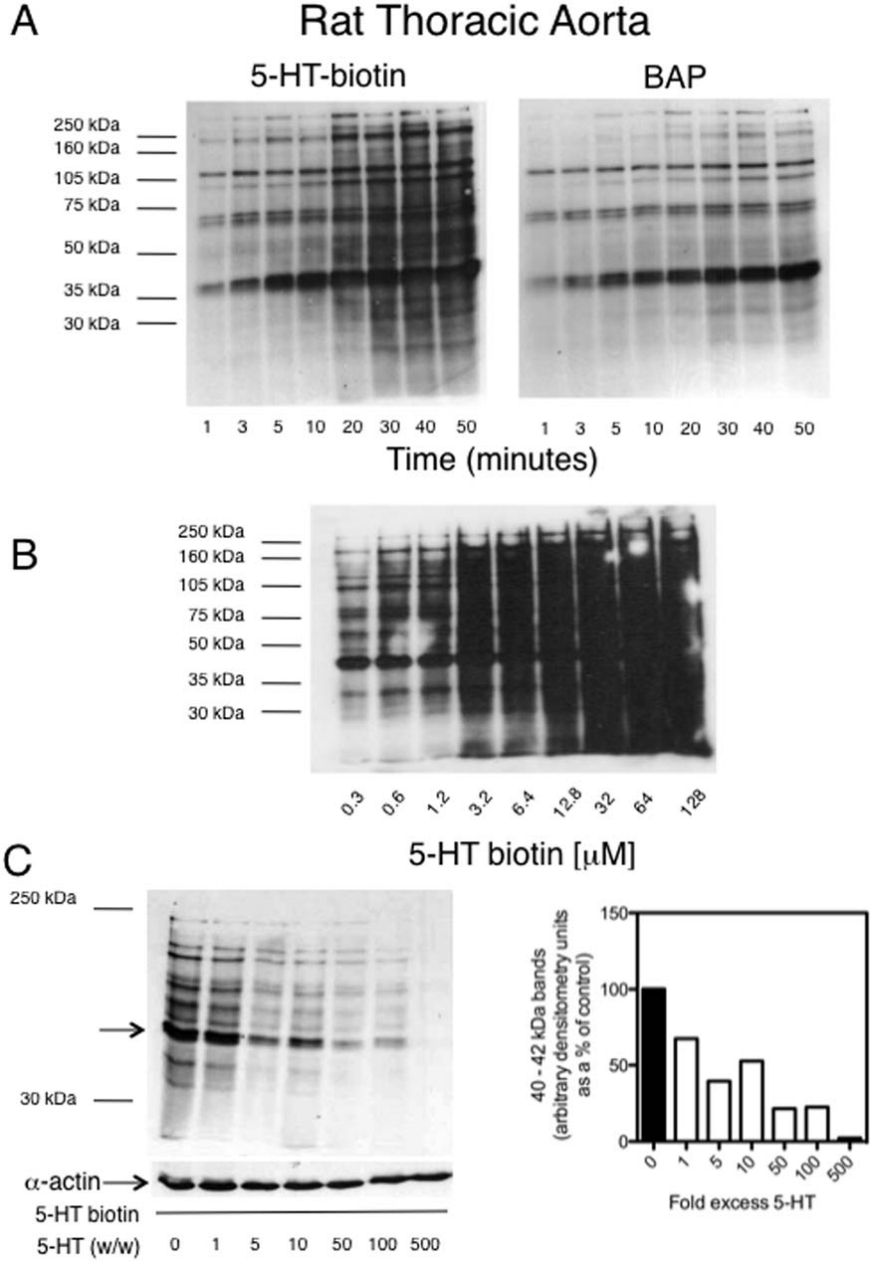
Protein serotonylation is time- and concentration-dependent in aortic homogenates and can be competed against by excess 5-HT. A. Time course of protein modification for 5-HT-biotin and BAP as substrates. Representative of N = 3 separate experiments. B. Concentration-response curve for aortic protein serotonylation. Representative of N = 5 separate experiments. C. Left: Ability of weight/weight excess of 5-HT to compete off 5-HT-biotin (12. 7 µM) in a TG reaction. Lower blot demonstrates that protein was loaded equally into all lanes as observed through equal α-actin expression. Right panel shows densitometry for the band at 40–42 kDa protein. Representative of N = 6 separate experiments.

Serotonylation of proteins was detected as early as 1 minute after addition of 5-HT-biotin (12.7 µM), with 5 bands (∼250–280, 220, 110, 70 and 40 kDa) being serotonylated rapidly and the process maximized by 30 minutes (**figure 3A**, compared to BAP as a substrate, 8 mM). Protein serotonylation was saturable, and serotonylation of proteins occurred at 5-HT concentrations that circulate in normal conditions (1 hour incubation, **figure 3B**). Serotonylation of proteins was inhibited by adding an excess of 5-HT (weight/weight) to the reaction mixture (**figure 3C** **, left**). The right panel of **figure 3C** shows densitometry for the 40–42 kDa band in the left figure (arrow) in the absence (control, 0) or presence of excess 5-HT. Serotonylation of any protein was not antagonized by the 5-HT_2A_ receptor antagonist ketanserin (100 nM, not shown).

### Serotonylation occurs in whole cells and is important to tissue function

**Figure 4:**
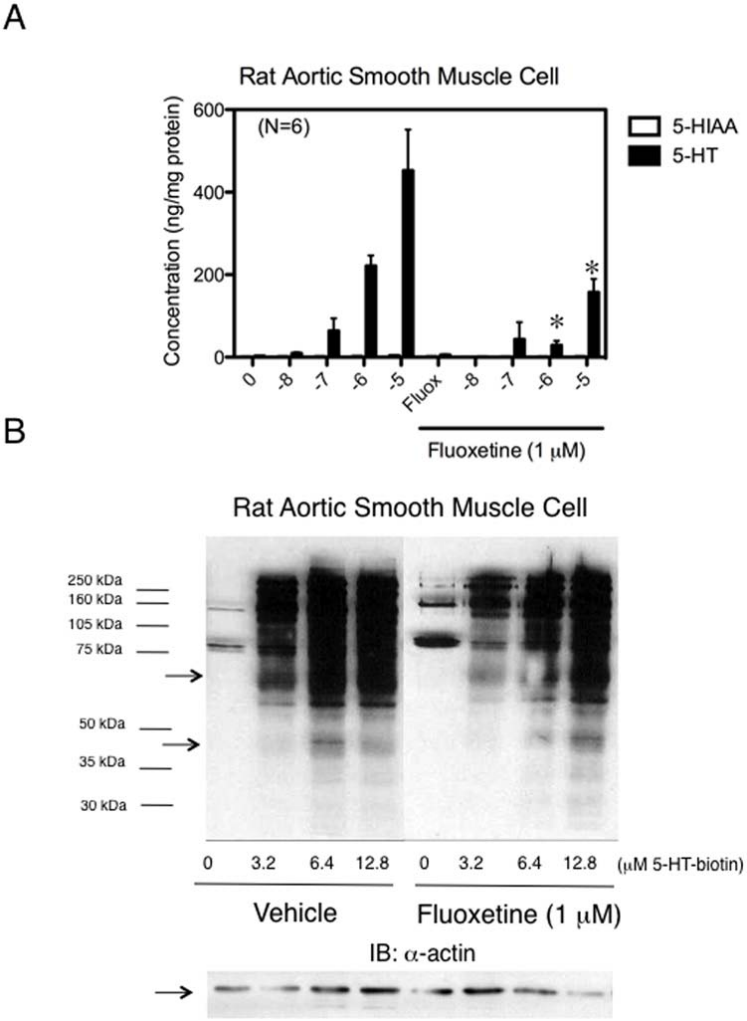
Aortic smooth muscle cells take up 5-HT and 5-HT-biotin; 5-HT-biotin is incorporated into cellular proteins. A. 5-HT uptake of rat aortic smooth muscle cells in the presence of the serotonin transporter inhibitor fluoxetine (1 µM). 5-HIAA levels were nearly zero, and are thus not visible but are marked on the graph. Data are from N = 6 separate aortic explants. Bars and vertical lines represent means±SEM. B. Serotonylation of cytosolic proteins upon incubation of 5-HT-biotin with rat aortic vascular smooth muscle cells in the presence and absence of the serotonin transporter inhibitor fluoxetine (1 µM); lower blot is α-actin loading control. Representative of cells from 6 different aortic explants.

Intact cells were capable of taking up 5-HT, and utilizing 5-HT in TG reactions. **Figure 4A** shows the results of 5-HT uptake studies in which cultured aortic smooth muscle cells were first incubated with the monoamine oxidase inhibitor pargyline (10 µM) to reduce 5-HT metabolism; 5-HIAA concentrations were near zero (open symbol). Cells were then exposed to increasing concentrations of 5-HT in the absence and presence of the serotonin transporter inhibitor fluoxetine (1 µM). Exogenous 5-HT was highly concentrated by the cells in a transporter-dependent manner. 5-HT-biotin was also taken up by the cells, with resultant concentration-dependent serotonylation of proteins (**figure 4B**). Serotonylation of some proteins was reduced (using the 3.2 µM 5-HT-biotin concentration, 75 and 40–42 kDa proteins (arrows) by ∼28% as ascertained through densitometry; also compare general density of 6.4 µM concentration+/−fluoxetine) but not abolished by the serotonin transporter inhibitor fluoxetine.

### Tandem mass spectrometry identifies α-actin as a serotonylated protein

Tandem mass spectrometry was used to identify the major proteins serotonylated. For these experiments, TG reactions were carried out normally in samples with and without 5-HT-biotin and then processed as described for tandem mass spectrometry (Materials and Methods). Five proteins were identified in every run of three different experiments, and those reported were only identified in reactions incubated with 5-HT-biotin. Smooth muscle α-actin (∼42 kDa), β-actin (∼41 kDa), γ-actin (∼41 kDa), myosin heavy chain (∼223 kDa) and the actin-binding protein filamin A (∼281 kDa) were identified as the major serotonylated proteins. The next experiments focused on α-actin.

**Figure 5:**
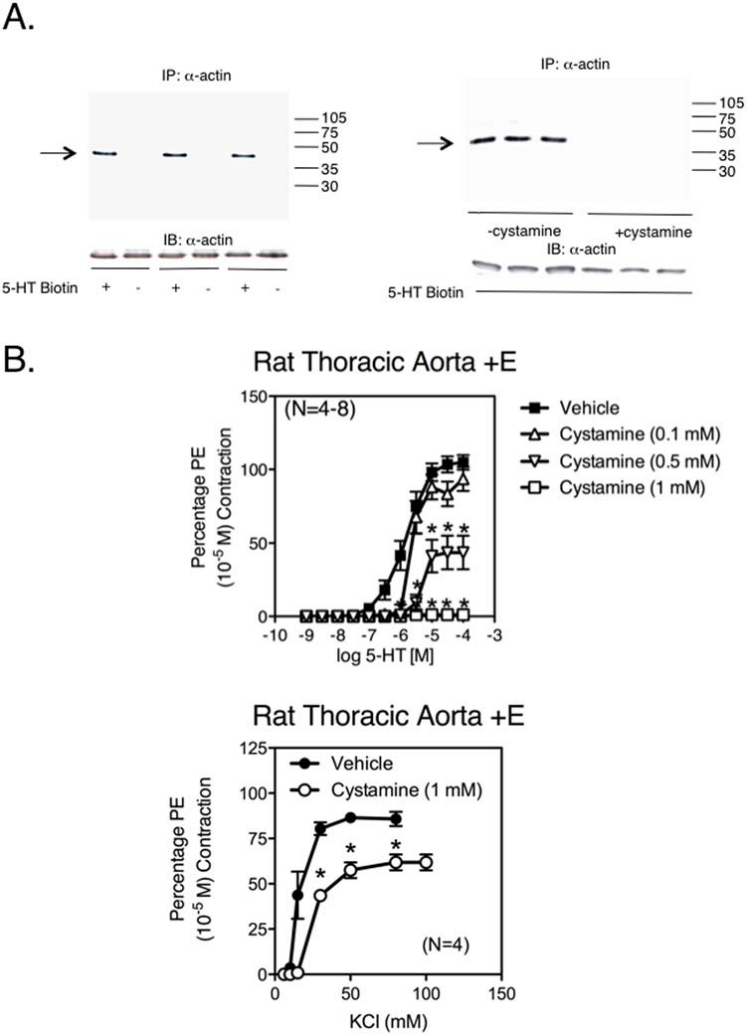
α-actin is serotonylated in aortic smooth muscle cells and inhibition of TG activity reduces aortic contraction to 5-HT. A. Immunoprecipitation of smooth muscle α-actin from rat aortic homogenates exposed to 5-HT-biotin in a standard transglutaminase reaction. Blots were developed using a streptavidin secondary (top), or exposed to a primary antibody against α-actin (bottom) and developed using standard horseradish peroxidase secondary antibody. Representative of N = 6 different experiments. B. Effect of vehicle (filled symbol) and cystamine (0.1–1 mM; open symbol) on 5-HT (top) and KCl (bottom)-induced contraction in isolated rat aorta. * indicates statistical difference from vehicle-incubated values. Points and vertical lines represent means±SEM for number of animals in parentheses.

Use of a streptavidin-linked HRP conjugated secondary antibody in blots of samples immunoprecipitated with smooth muscle α-actin validated that 5-HT-biotin was incorporated into α–actin, a process that was abolished by the TG inhibitor cystamine (**figure 5A**). Smooth muscle α-actin was present in all samples. The physiological relevance of serotonylation is supported by the abolishment of 5-HT-induced aortic contraction by cystamine in a concentration-dependent manner (**figure 5B** **, top**). By contrast, KCl-induced contraction was not abolished but was modestly reduced by cystamine (**figure 5B** **, bottom**; % PE contraction: vehicle = 85.8±4.00%; cystamine = 61.9±4.41%, p<0.05). In these same tissues, maximal contraction to PE (10 µM) was reduced by ∼80%.

**Figure 6:**
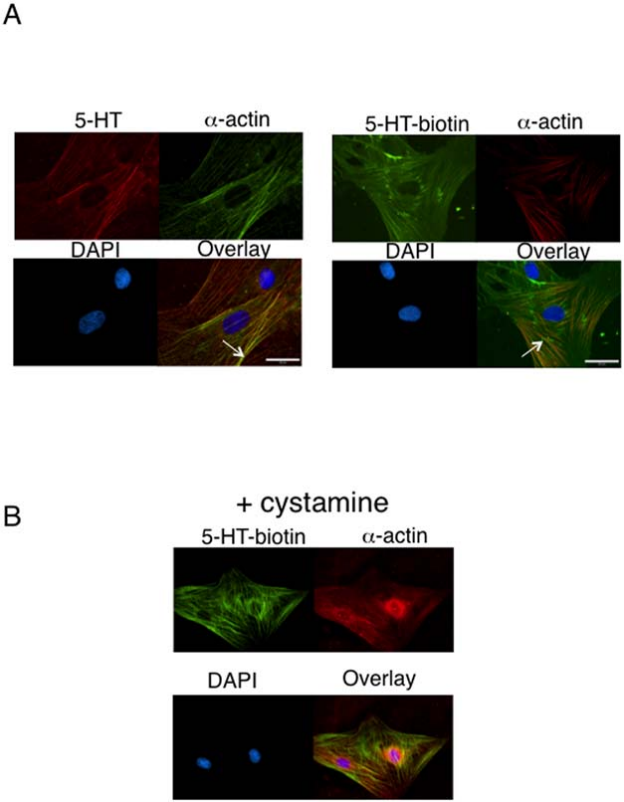
5-HT and 5-HT-biotin localize to α-actin and are incorporated into proteins. A. Immunocytochemistry of aortic smooth muscle cells incubated with exogenous 5-HT (12.7 µM; left) or 5-HT biotin (12.7 µM; right) and α-actin for 1 hour prior to fixation and visualization using an antirabbit fluorescent secondary (for 5-HT) or streptavidin-conjugated secondary (for 5-HT biotin). Representative of four different aortic explants. B. Effect of cystamine (10 mM) on 5-HT-biotin localization in aortic smooth muscle cells. Representative of four different aortic explants.

Two other lines of evidence support protein serotonylation. **Figure 6A** demonstrates that when cultured aortic smooth muscle cells are exposed to either 5-HT (left) or 5-HT-biotin (right) exogenously, the molecule is incorporated into the cell and colocalizes with smooth muscle α-actin. Presence of the TG inhibitor cystamine reduced the incorporation of 5-HT-biotin into actin, and disrupted α-actin filamentation (observed as loss of straight fibers, **Figure 6B**). The highest concentration of cystamine used (10 mM, 1 hour incubation in PSS) modestly but significantly reduced the viability of cells as assessed by trypan blue exclusion assay (69.0±3.2%, 2 explants, in duplicate), compared to cells treated with PSS without cystamine (92±2.5%).

### Serotonylation occurs in non-vascular tissues

**Figure 7:**
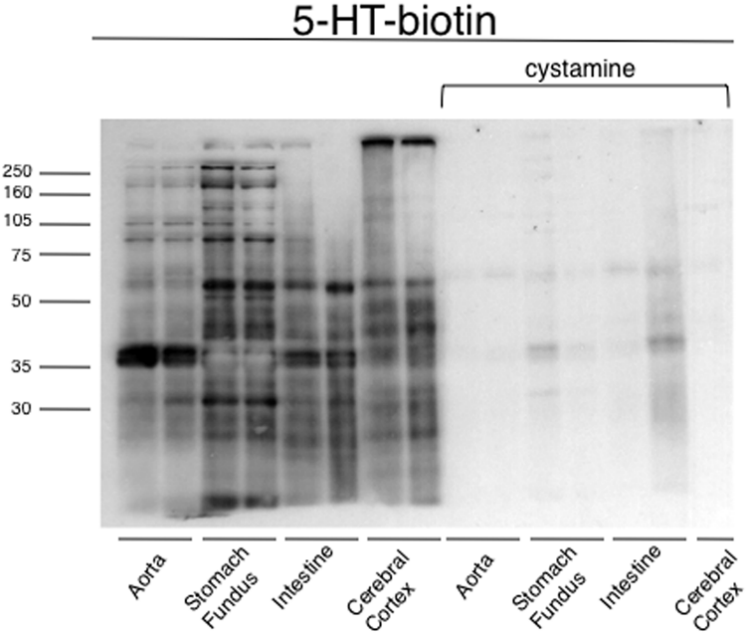
Serotonylation occurs in multiple tissues, smooth muscular and non-smooth muscular. Serotonylation of proteins from homogenates of rat thoracic aorta, rat stomach fundus, rat small intestine and rat cerebral cortex. The right half of the blot shows inhibition of serotonylation by the TG inhibitor cystamine. Representative of three separate experiments, each using 2 samples from different animals.

The final experiments tested whether serotonylation was unique to vascular tissue. We compared incorporation of the same concentration of 5-HT biotin (12.7 µM) into equivalent amounts of total protein isolated from aorta, stomach fundus (non-vascular smooth muscle), intestine (non-vascular smooth muscle and 5-HT synthesizing tissue) and cerebral cortex (non-muscular). **Figure 7** demonstrates that all tissues actively incorporated 5-HT-biotin into proteins in a TG-dependent fashion, as cystamine inhibited serotonylation. The proteins serotonylated were not, however, identical, though the actin proteins at ∼40 kDa were clearly present in all smooth muscle-based tissues.

## Discussion

The biological effects of 5-HT are diverse and numerous, with 5-HT exerting an effect in every physiological system. The present work reveals a receptor-independent mechanism utilized by 5-HT to modify vascular function, namely uptake of 5-HT and covalent modification of proteins that are involved in contraction. This work is important because of the implication of 5-HT in vascular diseases that include hypertension [systemic and pulmonary; 10], [11] and atherosclerosis [12].

### Serotonylated proteins

Confirming other reports, TG was present and functional in arterial tissue, both in homogenates and in aortic smooth muscle cells [13], [14]. Multiple proteins were serotonylated upon incubation with 5-HT-biotin, and this process was independent of 5-HT receptor interaction as inhibition of the receptor primarily expressed and active in the aorta, the 5-HT_2A_ receptor, did not modify protein serotonylation. While other 5-HT receptors are expressed in the aorta, they play a minimal role in contraction, and we have made the assumption that they would also play a minimal role in serotonylation. The proteins identified through mass spectrometry as being serotonylated share the common function of being involved, directly or indirectly, in contractility. It is reasonable to argue that these particular proteins—the actins, filamin A and myosin heavy chain– are serotonylated because they are among the most abundant in a smooth muscle-rich tissue. Chowdhury has demonstrated the association of TG with cellular stress fibers [15], placing all the elements for serotonylation close together. This idea is supported by the comparative blots in the smooth muscle-rich stomach fundus and intestine, in which the pattern of protein serotonylation is generally similar. This contrasts with the cortex. While cytoskeletal proteins such as β and γ actin are likely vital to many cellular functions in cortical tissue, smooth muscle α-actin is not heavily expressed. Other proteins are serotonylated in the cortex, evidenced by the different banding pattern of the TG reaction (**figure 7**). These findings suggest protein serotonylation is not specific to vascular smooth muscle.

For serotonylation to occur, intracellular 5-HT must exist. Arterial smooth muscle has two sources of 5-HT. First, arteries possess the serotonin transporter (SERT) and are able to take up exogenous 5-HT when it is released from 5-HT rich platelets [5]. Importantly, we presently demonstrated the ability of aortic smooth muscle cells to take up and concentrate 5-HT in a SERT-dependent fashion (**figure 4**). SERT is important for arterial tissue to take up exogenous 5-HT, but SERT is not solely responsible for uptake of 5-HT into tissues and cells. Arteries from SERT knockout rats continue to take up 5-HT, though in a diminished capacity [16]. Alternative means of 5-HT uptake is also one explanation for the inability of fluoxetine to abolish protein serotonylation in aortic smooth muscle cells (**figure 4**). Uptake, through multiple sources, is thus one way intracellular 5-HT can be enriched. Second, arteries have the ability to synthesize 5-HT [4]. Thus, both exogenous and endogenous 5-HT could potentially be used in serotonylation.

The presence of protein bands recognized by an antibody against 5-HT in samples that had not been exposed to either pargyline or exogenous 5-HT is important, because it suggests serotonylation of protein occurs in arterial smooth muscle exposed to endogenous 5-HT (internally synthesized or taken up). This is further supported by observations made in cultured smooth muscle. Normal serum, necessary to cellular growth, contains 5-HT (∼100 nM, estimated by HPLC). We routinely observe basal levels of endogenous serotonylation of proteins, and even serum-starving cells for 48 hours does not completely remove all serotonylated protein. Thus, one limitation of our cell experiments is that the anti-5-HT antibody we use cannot distinguish between 5-HT incorporated by incubation with media or our exogenously added 5-HT. One can speculate that because serotonylation may be irreversible, as are other TGII-dependent processes, serotonylation may be a signal for modifying the longevity of a protein in a cell. This idea will have to be investigated further. The important finding here is the ability of living cells to take up 5-HT actively and incorporate this 5-HT into proteins. Previous reports described 5-HT-derivatized proteins in 5-HT-rich platelets [17], [18], and our work demonstrates that serotonylation of proteins can occur in tissues relatively poor in 5-HT.

### Focus on α-actin and TG

The ability of 5-HT to associate with actin filaments has been known for over twenty years [19], [20], but this work is the first report to demonstrate direct modification of cytoskeletal/contractile proteins by 5-HT. Several lines of evidence support the association of 5-HT with α-actin, including incorporation of both 5-HT and 5-HT-biotin into α-actin protein, colocalization of 5-HT and 5-HT-biotin with α-actin in living smooth muscle cells and inhibition of 5-HT biotin placement on actin by the TG inhibitor cystamine. Isometric contraction in general and that elicited by 5-HT are α-actin-dependent processes [9]. 5-HT-induced isometric contraction was abolished by cystamine while contraction to KCl was modestly reduced. KCl was used in these experiments as a non-receptor dependent contraction, the mechanism of which is supported by L-type calcium channel activation and Rho Kinase [21], [22]. It was important to demonstrate that cells are functional in the presence of cystamine, and they are as evidenced by continued contraction to KCl. The inhibition of 5-HT-induced contraction by cystamine suggests that transglutaminase activity is crucial to 5-HT-induced contraction, perhaps by influencing serotonylation of proteins critical to contraction. However, given the effects of cystamine on KCl-induced contraction and contraction to a maximal concentration of phenylephrine, cystamine is likely influencing the function of contractile elements independent of the process of serotonylation. For example, TGII can also function as a G protein, named G_h_, and thus can serve contractility in a more global manner [23]. We have not been able to obtain TG2 knockout mice to separate out the role of TG in mediating 5-HT *vs* general agonist-stimulated arterial contractility. TG2 knockout mice are viable, and are suggested to be so by increased activity of TG1.

It is a goal to examine other TG inhibitors in contractility assays, but ones examined other than cystamine have questionable selectivity, and thus results from these experiments are difficult to interpret. Cystamine is not without problems, as trypan blue exclusion experiments suggested that high concentrations (10 mM) reduced cell viability by approximately 24%. Thus, it would be ideal to have additional inhibitors. We have tested dansylcadaverine (also a substrate for TG) as a TG inhibitor, and it abolishes both 5-HT and KCl-induced maximum contraction (data not shown). Dansylcadaverine has been published as a substance that can modify receptor internalization as well as inhibit TG activity [24]. Because of these many potential activities, it is difficult to interpret the results from experiments in which dansylcadaverine is used. High levels of guanosine triphosphate (GTP) have been reported to inhibit TG activity, but high GTP would activate any number of other signalling processes [25]. Small molecular inhibitors of TG are being developed but are not readily available [26], [27]. Thus, tools to continue this work should become available in the near future and will be valuable in addressing many of the important issues addressed here.

TG II, the isoform we observed to be expressed in arteries, is ubiquitous and calcium-dependent [23], [28], [29]. To be fair, we did not test for any of the other isoforms of TG reported [23]. TG, in general, has been implicated in the important vascular events of remodelling and Rho activation [30]–[35]. Cystamine exerts a largely irreversible inhibition against TG [36]. The functions of TG are themselves considered irreversible and mechanisms that reverse the effects of TG are unknown. Thus, it is presently unclear how the process of serotonylation, as carried out by TG, is regulated. Is serotonylation a terminal event for a protein, or can this be undone? Importantly, mechanisms of regulation for TG activity and expression are known, including the ability of nitric oxide and guanine nucleotides to modify TG function [37]–[39]. Thus, acute regulation of the serotonylating enzyme are recognized.

### Perspectives and Limitations

There are a number of limitations to this study that need to be recognized. First, we used a conduit artery for a majority of this work. We cannot directly apply our findings to how 5-HT, through TG-dependent mechanisms, plays a role in control of total peripheral resistance, an event largely supported by the smaller resistance arteries. Second, it has been difficult to demonstrate acute effects of 5-HT-biotin/5-HT on actin filamentation, structure or dynamics in the culture system given the synthetic nature of the cells. We have also been unable to completely remove endogenous 5-HT in these cells and whole tissues (synthesized). Third, identification of the glutamine residues modified by 5-HT biotin/5-HT would be helpful in ultimately allowing for determination of how serotonylation changes protein function. All proteins that were serotonylated contain a significant number of glutamine residues (actins: ∼20/700 residues; filamin A = 65/2647 residues, myosin heavy chain = 129/1938; rat sequences from NCBI), and thus the different sites and combinations thereof that could be serotonylated are numerous. In a similar vein, there are other 5-HT mediated functions, in addition to directly stimulated contraction, that have yet to be addressed in terms of the role of TG-catalyzed protein serotonylation. These include the ability of 5-HT to act as a mitogen and to potentiate the contractile (and mitogenic) effects of other vasoactive substances. The latter is a particularly important function, as the mechanisms behind contractile potentiation/synergy stimulated by 5-HT are not well understood [40]. There is support for an intracellular process utilizing 5-HT to be important for vascular smooth muscle cell mitogenesis, as pulmonary arterial smooth muscle cells depend on the function of the serotonin transporter for 5-HT-stimulated mitogenesis [41], [42]. Finally, we have focused on α-actin modification by 5-HT, as understanding vascular smooth muscle function is central to our laboratory. We have yet to investigate the effects of serotonylation on the cytoskeletal proteins filamin A, β or γ actin. Filamin A is a large protein (281 kDa) that serves as an anchor for proteins like β and γ actin [43]. The significant serotonylation of these proteins suggests that 5-HT exerts a concerted effect on proteins involved in contraction and the cytoskeleton. Another potential avenue of interest for future work is how 5-HT modifies proteins that can modify 5-HT's biological effects and concentration. These include 5-HT receptors, SERT, and enzymes critical to the synthesis of 5-HT such as tryptophan hydroxylase. An understanding of whether and how 5-HT potentially feeds back to elements necessary to 5-HT signal transduction may provide new insight into the regulation of serotonergic systems.

In summary, these studies present the novel findings of serotonylation of contractile proteins in vascular smooth muscle by the enzyme TG. In particular, the covalent modification of smooth muscle α-actin by 5-HT supports that 5-HT influences arterial contractility in a receptor-independent manner. These findings suggest that primary amines like 5-HT may carry out biological effects in receptor-independent manners, and thus this work potentially extends to numerous other avenues of research.
